# Additional Genetic Alterations and Clonal Evolution of MPNs with Double Mutations on the *MPL* Gene: Two Case Reports

**DOI:** 10.3390/hematolrep15020033

**Published:** 2023-05-23

**Authors:** Maria Stella Pennisi, Sandra Di Gregorio, Elena Tirrò, Chiara Romano, Andrea Duminuco, Bruno Garibaldi, Gaetano Giuffrida, Livia Manzella, Paolo Vigneri, Giuseppe A. Palumbo

**Affiliations:** 1 Department of Clinical and Experimental Medicine, University of Catania, 95123 Catania, Italy; 2 Center of Experimental Oncology and Hematology, A.O.U. Policlinico “G. Rodolico-San Marco”, 95123 Catania, Italy; 3 Postgraduate School of Hematology, University of Catania, 95123 Catania, Italy; 4 Hematology Unit and Bone Marrow Transplant, A.O.U. Policlinico “G. Rodolico-San Marco”, 95123 Catania, Italy; 5 Department of Medical, Surgical Sciences and Advanced Technologies, “G.F. Ingrassia”, University of Catania, 95123 Catania, Italy

**Keywords:** essential thrombocythemia, primary myelofibrosis, MPL^V501A-W515L^, JAK2^V617F^, cis mutations

## Abstract

Essential thrombocythemia (ET) and primary myelofibrosis (PMF) are two of the main *BCR-ABL1*-negative chronic myeloproliferative neoplasms (MPNs) characterized by abnormal megakaryocytic proliferation. *Janus kinase 2* (*JAK2*) mutations are detected in 50–60% of ET and PMF, while *myeloproliferative leukemia* (*MPL*) virus oncogene mutations are present in 3–5% of cases. While Sanger sequencing is a valuable diagnostic tool to discriminate the most common MPN mutations, next-generation sequencing (NGS) is a more sensitive technology that also identifies concurrent genetic alterations. In this report, we describe two MPN patients with simultaneous double *MPL* mutations: a woman with ET presenting both *MPL*^V501A-W515R^ and *JAK2*^V617F^ mutations and a man with PMF displaying an uncommon double *MPL*^V501A-W515L^. Using colony-forming assays and NGS analyses, we define the origin and mutational landscape of these two unusual malignancies and uncover further gene alterations that may contribute to the pathogenesis of ET and PMF.

## 1. Introduction

Essential thrombocythemia (ET) and primary myelofibrosis (PMF) are two of the main *BCR-ABL1*-negative myeloproliferative neoplasms (MPNs). Over 70% of ET and PMF patients display a specific genetic alteration: 56% present *Janus Kinase 2* (*JAK2*) mutations, 20–25% exhibit *calreticulin* (*CALR*) alterations, and 3–5% show nucleotide substitutions in the *myeloproliferative leukemia* (*MPL*) virus oncogene [1,2,3].

JAK2 is a non-receptor tyrosine kinase that contributes to myeloid cell proliferation and differentiation [4,5]. When *JAK2* displays the common V617F mutation, the ensuing protein acquires constitutive catalytic activity even in the absence of cytokine stimulation [6]. CALR is an endoplasmic reticulum chaperone protein. The main mutations consist of insertions or deletions in exon 9, resulting in a positively charged C-terminus. The mutant CALR forms a stable complex with the thrombopoietin receptor (TPO-R) upregulating the JAK/STAT pathway [7]. The *MPL* gene encodes for TPO-R. Excessive TPO-R activation promotes megakaryocytic hyperplasia and bone marrow fibrosis [8,9]. The most frequently reported *MPL* mutation involves the W515 residue on exon 10 [10].

Double *MPL* mutations have been previously described but represent an extremely infrequent event [11,12,13,14,15,16]. Ma et al. identified an *MPL*^W515L^ mutation associated with the S505N substitution [12,13]. Pietra and colleagues detected double *MPL* mutations in three subjects: two ET patients, one presenting the S505C-W515L double mutation and the other with the V501A-W515R sequence alteration, and an individual with PMF displaying the V501A-W515L mutations [11]. However, the presence of additional genetic alterations possibly contributing to disease initiation and progression was not investigated [16,17,18]. Furthermore, these reports failed to establish if the observed genetic alterations were in cis (on the same allele) or in trans (on two different alleles) [11,19].

In this report, we describe two patients diagnosed with MPNs expressing two concurrent *MPL* mutations identified by Next Generation Sequencing (NGS) that were investigated for the presence of additional genetic alterations in order to discern the possible clonal evolution of their disease.

## 2. Case Presentation

### 2.1. Case 1

In May 2017, a 57-year-old woman presenting ischemic symptoms was admitted to the Hematology Unit of the A.O.U. Policlinico “G. Rodolico-San Marco” in Catania with a high platelet count (807 × 10^9^/L) (Table 1).

Following the World Health Organization (WHO) guidelines [20], the patient was assessed for the presence of *JAK2* and *CALR* mutations by Sanger sequencing (SS). In particular, the genomic DNA (gDNA) was extracted from 1.7 mL of peripheral blood (PB) using the Qiasymphony DSP DNA Midi kit (Qiagen), according to the manufacturer’s protocol. For both analyses, we performed a Polymerase Chain Reaction (PCR) using 300 ng of gDNA. To amplify exon 14 of JAK2, we employed the forward (FW) primer 5′-GGGTTTCCTCAGAACGTTGA-3′ and the reverse (RV) primer 5′-TCATTGCTTTCCTTTTTCACAA-3′. To amplify exon 9 of CALR, we employed the FW 5′-CCTGCAGGCAGCAGAGAAAC-3′ and the RV 5′-ACAGAGACATTATTTGGCGCG-3′ primers. The PCR conditions consist of an initial denaturation step at 95 °C for 5 min, then 35 cycles of denaturation (95 °C for 45 s), annealing (57 °C for 30 s for *JAK2*; 56.5 °C for 30 s for *CALR*), extension (72 °C for 30 s), and a final extension at 72 °C for 7 min. The final PCR products (460 bp for JAK2 and 288 bp for CALR) were loaded on an agarose gel in TAE 1.2%, purified, and sequenced by SS using the same FW primers employed for the PCR. Both sequencing results were negative for the presence of mutations in the two genes.

**Figure 1:**
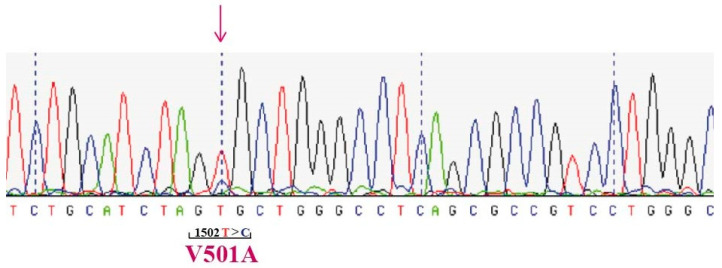
Electropherogram obtained by Sanger sequencing of the *MPL* gene obtained by DNA extracted from the peripheral blood of patient 1.

Subsequently, we tested the gDNA of the patient for the presence of *MPL* mutations on exon 10. In particular, 700 ng of gDNA were employed to perform a PCR using the FW 5′-AGTAGGGGCTGGCTGGATGA-3′ and the RV 5′-TGCCTGTTTACAGGCCTTCG-3′ primers. The Hot Start PCR conditions consist of an initial denaturation step at 94 °C for 5 min, then 80 °C for 1 min, followed by 35 cycles of denaturation (94 °C for 30 s), annealing (60.2 °C for 50 s), extension (72 °C for 30 s), and a final extension at 72 °C for 10 min. The final PCR product of 235 bp was loaded on an agarose gel in TAE 1.2%, purified, and sequenced by SS using the same FW primer employed for the PCR. Sequencing of the *MPL* gene revealed a V501A (c.1502T>C) substitution (Figure 1).

The patient underwent a bone marrow (BM) aspiration that revealed megakaryocytic hyperplasia and grade 1 fibrosis. Eight months later (February 2018), she still presented a high platelet count (931 × 10^9^/L). A second BM biopsy confirmed her ET diagnosis. Spleen size was normal.

To better analyze the molecular profile of this patient, we performed an NGS analysis. The sensitivity of NGS is much greater than that of SS, as it is able to detect mutant alleles with a frequency lower than 1%, while SS has a limit of detection around 20%. We analyzed the DNA isolated from the patient’s PB and BM, employing the Ion Ampliseq Cancer Hotspot Panel v2 (Thermo Fisher Scientific, Waltham, MA, USA) as previously reported [21]. This NGS panel consists of 207 primer pairs covering approximately 2.800 COSMIC (Catalogue of Somatic Mutations in Cancer) mutations from 50 oncogenes and tumor suppressor genes. Sequences were analyzed with the Ion Reporter software version 5.16 (Thermo Fisher Scientific). Variants with coverage lower than 100× and an allele frequency (AF) lower than 5% were filtered out. In the PB sample, our analysis identified both the V501A and the W515R mutations, the latter not detected by SS since it was expressed with a variant allele frequency (VAF) < 20% (14.36%) (Table 2). The NGS analyses performed on the BM confirmed both MPL mutations with a VAF of 100%. We also detected 5 additional hotspot mutations: *JAK2*^V617F^ (VAF 39.77%), *PTEN*^Q17H^ (VAF 27.88%), *TP53*^H178Tfs*69^ (VAF 11.5%), *PIK3CA*^N1044D^ (VAF 6.02%), and *KIT*^N566T^ (VAF 1.62%) (Table 2). The relatively low VAF of the *JAK2* mutation (39.77%) might explain why this alteration was not detected in her PB, as it may have been expressed in a limited number of neoplastic clones in the PB.

### 2.2. Case 2

In October 2018, a 68-year-old man was referred to the Hematology Unit of the A.O.U. Policlinico “G. Rodolico-San Marco” in Catania with high white blood cell (102 × 10^9^/L) and platelet (785 × 10^9^/L) counts (Table 3).

**Figure 2:**
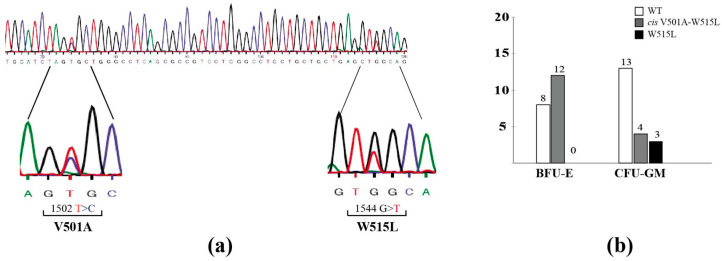
Sanger sequencing and colony-forming assays for the identification of MPL mutations. (**a**) Electropherogram obtained by Sanger sequencing of the *MPL* gene obtained by DNA extracted from the peripheral blood of patient 2. (**b**) Histogram indicating the distribution of BFU-Es and CFU-GMs obtained from the BM biopsy of patient 2: wild-type (WT) colonies are shown in white; colonies within the *cis*-double MPL^V501A-W515L^ mutation are in gray; colonies with the single W515L mutation are in black.

He was promptly subjected to mutational screening by SS for the expression of the JAK2^V617F^ substitution, but the test was negative as it was for CALR mutations. Subsequent analyses for additional genetic alterations detected the presence of two mutations on the MPL gene: V501A (c.1502T>C) and W515L (c.1544G>T) (Figure 2a). The BM biopsy showed granulocyte hyperplasia and megakaryocyte clustering with moderate (grade 1) fibrosis, and, on this basis, the diagnosis of early PMF was formulated. A mild splenomegaly was detected (longitudinal diameter: 13 cm).

To establish if the detected double mutations were in *cis* or in *trans*, we carried out the clonal selection of Burst-Forming Units-Erythroid (BFU-E) and Colony-Forming Units-Granulocyte and Monocyte (CFU-GM), followed by SS on the DNA extracted from these colonies. In detail, 1 × 10^5^ primary mononuclear cells (MNCs) were isolated from BM biopsies by density gradient centrifugation. For the colony-forming assay, MNCs were plated at a density of 10.000 cells/mL (in triplicate) in Methocult H4435 (StemCell Technologies, Vancouver, B.C., Canada) and incubated at 37 °C in 5% CO_2_ for 12 days. Forty individual colonies (20 BFU-E and 20 CFU-GM) were plucked and transferred into PCR tubes for DNA extraction by thermic lysing (94° for 10 min). Subsequently, DNA was subjected to the same PCR described above for the amplification of MPL exon 10 and sequenced by SS [22]. Twelve out of 20 BFU-E colonies carried the heterozygous *cis* V501A-W515L mutations, while the remaining 8 exhibited the wild-type gene (Figure 2b). Of the 20 CFU-GM colonies, only 4 were positive (in *cis*) for the double mutation, while the remaining 16 were wild-type (13 colonies) or only expressed the W515L substitution (3 colonies) (Figure 2b). These data suggest a more significant heterogeneity in CFU-GM compared to BFU-E colonies and the existence of two distinct clones carrying different mutations (single or double in *cis*) [23].

To further analyze the genetic profile of the disease and understand its possible clinical evolution, we performed an NGS analysis on the DNA obtained from the PB of the patient, employing the same procedures described above. Sequencing data confirmed both the V501A and the W515L mutations with a VAF of 38.5% and 34.4%, respectively. The analysis also detected the KIT^M541L^ mutation (VAF 59.7%), frequently reported in mastocytosis and chronic eosinophilic leukemia [3,24,25] (Table 4).

## 3. Discussion

In the setting of myeloproliferative diseases, knowledge about gene mutations increasingly plays a leading role. In all the newly developed prognostic models, unfavorable mutations in several genes are considered central to establishing the prognosis [26]. Gene analysis has become a widely used procedure that has made it possible to identify cases of concurrent alterations, possibly also for the same gene. In this setting, double mutations in the *MPL* gene are extremely uncommon in patients affected by *BCR-ABL1*-negative MPNs [11,12,13]. In our cohort of patients, diagnosed from 2012 to date, the frequency of MPL mutations is around 3% (44 out of 1244 total patients), and of these, only the two patients reported in this work presented a double mutation on the gene. Likewise, concurrent mutations in *JAK2* and *MPL* are rare but can co-exist in subjects with ET or with myelofibrosis exhibiting myeloid metaplasia [27,28]. Indeed, the first case reported in this paper is the only one in our cohort.

Sanger sequencing and NGS are currently used to investigate the mutational profile of MPN patients. Sanger sequencing is the least sensitive technique and can discern double mutations only if they are expressed at or above the 20% threshold. However, SS may help discriminate between cis and trans mutations after clonal selection of single BFU-E or CFU-GM colonies. On the contrary, NGS analyzes multiple genetic regions with a sensitive limit of detection (i.e., <1%) and high accuracy, discerning nucleotide variants, small insertions and deletions, copy number variations, and fusion transcripts [29].

We report an ET patient simultaneously carrying the V501A and the W515R mutations. Next-generation sequencing analysis of her BM biopsy also detected a *JAK2^V617F^* alteration. The lower frequency of the *JAK2* substitution (39.77%) in the BM compared to *MPL* (100%) might explain the absence of the former alteration in the patient’s PB and suggest progressive disease evolution from a pre-existing dominant *MPL*-mutated clone [30]. The second case concerns a PMF patient with a rare *MPL^V501A-W515L^* double mutation, likely generated in *cis* by a hematopoietic precursor mostly differentiating along the erythroid lineage. While the *MPL^W515L^* mutation is known to promote EPO-independent activation of the receptor [31], the pathogenic role of the V501A substitution is still unclear. Recently, Bridgford reported that the non-canonical V501A MPL mutation causes cytokine-independent growth in Ba/F3 cells [32], which could contribute to the disease’s pathogenesis.

To date, the presence of numerous (ET patient) or limited (PMF patient) additional genetic alterations has not yet translated into a more aggressive disease, as both patients are currently in excellent clinical conditions while only receiving 100 mg daily acetylsalicylic acid for thromboembolic event prophylaxis, with a follow-up of more than 5 years since diagnosis. However, the employment of a new commercially available NGS panel specific for MPNs or myelodysplastic syndromes could be of great interest to explore other novel genetic and/or epigenetic mechanisms involved in the pathogenesis of the disease. Indeed, we recently extended NGS analysis in MPNs using a DNA/RNA panel including 40 genes and 29 fusion transcripts involved in myeloid malignancies. This new NGS panel is of great interest as it is able to confirm the diagnosis and discriminate genetic profiles involved in clonal evolution and disease progression of MPN/MDS.

In conclusion, NGS analyses coupled with colony-forming assays allow the characterization of the molecular landscape and clonal evolution of MPN patients with uncommon genetic mutations. A longer follow-up will be required to address the prognostic impact of this information.

## Figures and Tables

**Table 1:** Clinical data of patient 1 at diagnosis.

Clinical Data of Patient 1	
Age	57
Gender	female
Platelet count	807 × 10^9^/L
Hemoglobin level	12.8 g/dL
Red blood Cells (RBC)	4.13 × 10^9^/L
White blood Cells (WBC)	6.58 × 10^9^/L
Liver and spleen dimension	N.I.
Other diseases	ischemic colitis, splenic aneurysm, and retinal vascular occlusion

**Table 2:** Hotspot mutations detected by NGS in the specified samples of patient 1.

Gene	Coding	Protein	Cosmic ID	Type of Mutation	VAF%	Fathmm Prediction Score
DNA from peripheral blood						
*MPL*	c.1502T>C	p.V501A	COSM86964	Substitution-Missense	27.36	Neutral (0.40)
*MPL*	c.1543T>A	p.W515R	COSM29008	Substitution-Missense	14.36	Pathogenic (0.54)
DNA from bone marrow						
*MPL*	c.1502T>C	p.V501A	COSM86964	Substitution-Missense	100	Neutral (0.40)
*MPL*	c.1543T>A	p.W515R	COSM29008	Substitution-Missense	100	Pathogenic (0.54)
*JAK2*	c.1849G>T	p.V617F	COSM12600	Substitution-Missense	39.77	Pathogenic (0.94)
*PTEN*	c.51A>C	p.Q17H	Novel	Substitution –Missense	27.88	-
*TP53*	c.532delC	p. H178Tfs*69	COSM43978	Deletion Frameshift	11.5	n/a
*PIK3CA*	c.3130A>G	p.N1044D	COSM27134	Substitution-Missense	6.02	Pathogenic (0.96)
*KIT*	c.1697A>C	p.N566T	COSM9233350	Substitution-Missense	1.62	Pathogenic (0.97)

**Table 3:** Clinical data of patient 2 at diagnosis.

Clinical Data of Patient 2	
Age	68
Gender	male
Platelet count	785 × 10^9^/L
Hemoglobin level	12.5 g/dL
Red blood Cells (RBC)	4.25 × 10^9^/L
White blood Cells (WBC)	102 × 10^9^/L
Liver and spleen dimension	mild splenomegaly
Other diseases	hypertension and diabetes mellitus

**Table 4:** Hotspot mutations detected by NGS in the DNA from the peripheral blood of patient 2.

Gene	Coding	Protein	Cosmic ID	Type of Mutation	VAF%	Fathmm Prediction Score
*MPL*	c.1502T>C	p.V501A	COSM86964	Substitution-Missense	38.5	Neutral (0.40)
*MPL*	c.1544G>T	p.W515L	COSM18918	Substitution-Missense	34.4	Pathogenic (0.70)
*KIT*	c.1621A>C	p.M541L	COSM28026	Substitution-Missense	59.7	Pathogenic (0.74)

## Data Availability

Data are available from the authors upon request.
